# Intermittent Hypoxia Mediates Paraspeckle Protein-1 Upregulation in Sleep Apnea

**DOI:** 10.3390/cancers13153888

**Published:** 2021-08-02

**Authors:** Elena Díaz-García, Sara García-Tovar, Raquel Casitas, Ana Jaureguizar, Ester Zamarrón, Begoña Sánchez-Sánchez, Ana Sastre-Perona, Eduardo López-Collazo, Francisco Garcia-Rio, Carolina Cubillos-Zapata

**Affiliations:** 1 Grupo de Enfermedades Respiratorias, Instituto de Investigación Sanitaria del Hospital Universitario La Paz (IdiPAZ), 28029 Madrid, Spain; elena.diaz.garcia@idipaz.es (E.D.-G.); sarugarto@gmail.com (S.G.-T.); 2 Centro de Investigación Biomédica en Red en Enfermedades Respiratorias (CIBERES), 28029 Madrid, Spain; rqlkas@yahoo.es (R.C.); anajaureguizaroriol@gmail.com (A.J.); elcollazo@hotmail.com (E.L.-C.); 3 Servicio de Neumología, Hospital Universitario La Paz, 28029 Madrid, Spain; ester.zamarron@gmail.com (E.Z.); bsanchezsanchez@salud.madrid.org (B.S.-S.); 4 Servicio de Neumología, Hospital Universitario Ramón y Cajal, 28034 Madrid, Spain; 5 Grupo deTerapias Experimentales y Biomarcadores en Cáncer, Instituto de Investigación Sanitaria del Hospital Universitario La Paz (IdiPAZ), 28029 Madrid, Spain; ana.sastre.perona@idipaz.es; 6 Grupo de Respuesta Inmune Innata, Instituto de Investigación Sanitaria del Hospital Universitario La Paz (IdiPAZ), 28029 Madrid, Spain; 7 Facultad de Medicina, Universidad Autónoma de Madrid, 28029 Madrid, Spain

**Keywords:** paraspeckle component 1, intermittent hypoxia, HIF1α, sleep apnea, TGFβ

## Abstract

**Simple Summary:**

Patients with obstructive sleep apnea (OSA) exhibit an intermittent hypoxia-dependent paraspeckle protein-1 (PSPC1) increase, which is eventually delivered to the plasma through its cleavage from OSA monocytes by matrix metalloprotease-2, promoting tumor growth factor (TGFβ) expression and increasing epithelial-to-mesenchymal transition in a tumor functional model using a melanoma cell line. These results connect the phenomena of sleep apnea with increased plasma PSPC1 levels, which has a functional effect on the TGFβ pathway and accelerates tumor progression.

**Abstract:**

As some evidence suggests that hypoxia might be an inducer of nuclear paraspeckle formation, we explore whether intermittent hypoxia (IH)-mediated paraspeckle protein-1 (PSPC1) overexpression might contribute to the activation of tumor growth factor (TGF)β-SMAD pathway in patients with obstructive sleep apnea (OSA). This activation would promote changes in intracellular signaling that would explain the increased cancer aggressiveness reported in these patients. Here, we show that patients with OSA exhibit elevated PSPC1 levels both in plasma and in monocytes. Our data suggest that PSPC1 is ultimately delivered to the plasma through its cleavage from OSA monocytes by matrix metalloproteinase-2 (MMP2). In addition, IH promotes PSPC1, TGFβ, and MMP2 expression in monocytes through the hypoxia-inducible factor. Lastly, both PSPC1 and TGFβ induce increased expression of genes that drive the epithelial-to-mesenchymal transition. Our study details the mechanism by which hypoxemia upmodulates the extracellular release of PSPC1 by means of MMP2, such that plasma PSPC1 together with TGFβ activation signaling further promotes tumor metastasis and supports cancer aggressiveness in patients with OSA.

## 1. Introduction

Increasing information has recently emerged about the potential associations between obstructive sleep apnea (OSA), cancer aggressiveness, and mortality [1,2,3]. Both in OSA patients as well as in vitro and animal models [4,5,6], hypoxia-inducible factor (HIF1α) overexpression secondary to intermittent hypoxia (IH) compromises the immunosurveillance system by altering several immune subsets, which favors the development of a tumor-promoting environment [5,6,7]. The HIF1α-mediated induction of transforming growth factor β (TGF-β) appears to play a key role in establishing an immunosuppressive phenotype in the monocytes and natural killer cells of patients with OSA [6]. Interestingly, TGFβ is abundantly expressed in the tumor microenvironment and is known to play pleiotropic roles in cancer progression [8] and in the development of metastasis, a multistep process that requires cancer cells with flexible self-reprogramming capabilities to transition from the epithelial to the mesenchymal state (EMT) and to achieve cancer stem cell (CSC)-like features for surviving attacks from apoptotic signals [9,10]. At tumor initiation, TGFβ might act as a tumor suppressor, inducing apoptosis [11]. In late tumor stages, however, high TGFβ levels lead to tumor metastasis [12,13], mainly by driving EMT [14,15]. In particular, the TGFβ pathway promotes EMT by activating the transcriptional factors SNAIL and SLUG, which suppress the expression of epithelial markers, such as E-cadherin, while inducing the expression of other EMT-related transcription factors, such as TWIST [16,17,18,19,20]. The dichotomous role of TGFβ has been attributed to its forms (activated and non-activated) and to the differences in the cellular context, which determine TGFβ responses [15,21].

Paraspeckles are nuclear bodies located in the interchromatin space of the cell nucleus, adjacent to speckles [22]. These nuclear bodies are mainly composed of long non-coding RNA NEAT1 and three proteins, one of which is paraspeckle component 1 (PSPC1). Paraspeckle formation is dynamic and triggered by numerous cell stress scenarios, including malignant transformation. An animal model using multiple cancer cell types showed that PSPC1 correlates with poor survival, potentiating EMT and TGFβ signaling [23]. Data from a transcriptome and gene set enrichment analysis revealed that PSPC1 is the master modulator that activates the signature gene sets of EMT, CSC, and TGFβ signaling [24]. Indeed, PSPC1 expression upregulates EMT-transcription factors (TFs) (such as TWIST, SNAIL, and SLUG) and CSC-TF (OCT4, SOX2, and NANOG) [23,25]. Moreover, the PSPC1 effect is dependent on canonical TGFβ signaling because treatment with a TGF beta receptor 1 inhibitor (SB431542) abolishes PSPC1-enhanced expression of core EMT-TF and CSC-TF, leading to decreased cellular migration, invasion, and reduced CSC populations [25].

Although it has been proposed that PSPC1 upregulation in tumor cells could be due to cellular stress [26], its mechanism has not been completely clarified. However, evidence suggests that hypoxia might induce nuclear paraspeckle formation. Specifically, activation of LncRNA-NEAT1 (another architectural component of nuclear paraspeckles) in response to hypoxia has been reported [27]. In vitro studies have demonstrated that HIF1α regulates LncRNA-NEAT1 transcription, maintaining cancer cell growth and inhibiting their apoptosis and cell cycle arrest [28]. The potential contribution of hypoxia to PSPC1 expression could be relevant in tumor cells, given that hypoxia is a common characteristic of the tumor microenvironment. In turn, IH-dependent PSPC1 overexpression in patients with OSA might explain the TGFβ upregulation promoting the development of a baseline pro-tumoral state, which could lead to greater aggressiveness in developing tumors.

In this study, we explored whether IH-mediated PSPC1 overexpression might contribute to the activation of the TGFβ/SMAD pathway, promoting EMT and CSC and explaining the increased cancer aggressiveness in patients with OSA.

## 2. Materials and Methods

### 2.1. Study Participants

Patients with OSA were consecutively recruited from the sleep unit of La Paz-Cantoblanco University Hospital, Madrid, Spain. Fifty newly diagnosed patients with an apnea-hypopnea index (AHI) > 30 with no previous treatment were included in the study. The OSA diagnosis was established by respiratory polygraphy (Embletta GOLD, ResMed), which included the continuous recording of oronasal flow and pressure, heart rate, thoracic and abdominal respiratory movements, and oxygen saturation (SpO_2_). Those tests in which the patients claimed to sleep less than 4 h or in which there were less than 5 h of nocturnal recording were repeated. The exclusion criteria were as follows: previous or current treatment with oxygen or mechanical ventilation; underweight (body mass index [BMI] < 18.5 kg/m^2^); morbid obesity (BMI > 40 kg/m^2^); previous or current evidence of neoplastic disease; previous diagnosis of chronic obstructive pulmonary disease, asthma or respiratory failure; any infectious disease in the last 3 months; and treatment with inhaled or systemic corticosteroids or other anti-inflammatory drugs.

As a control group, healthy volunteers (HV) similar in sex, age, BMI, and smoking habits were selected. None of these volunteers were being treated with any type of medication, and OSA was ruled out by respiratory polygraphy.

The study was approved by the local ethics committee (PI-3620), and informed consent was obtained from all participants.

### 2.2. Peripheral Blood Mononuclear Cell Isolation and Culture

Peripheral blood mononuclear cells (PBMCs) were isolated from blood samples of the patients with OSA and HV using Ficoll-Paque Plus (Amersham Biosciences, Uppsala, Sweden) gradient by centrifugation. Isolated PBMCs were then cultured in Roswell Park Memorial Institute (RPMI) 1640 medium, which was supplemented with 100 U/mL of penicillin and 100 μg/mL of streptomycin, without fetal bovine serum, on an adherent surface for 1 h at 37 °C and 5% carbon dioxide (CO_2_) for monocyte enrichment. A total of 0.5 × 10^6^ monocytes per well were distributed in 6-well plates. The medium was subsequently changed with fresh RPMI 1640 medium containing 100 U/mL of penicillin, 100 μg/mL of streptomycin, and 10% fetal bovine serum. The cells were incubated at 37 °C and 5% CO_2_ overnight.

### 2.3. In Vitro Intermittent Hypoxia Model

In the IH models, monocytes from HV were seeded as described in the previous paragraph and cultured in an incubation chamber attached to an external oxygen/nitrogen computer-driven controller using BioSpherix OxyCycler C42 (Redfield, NY, USA), a system that generates periodic changes in oxygen concentrations and controls air gas levels in each chamber, while individually maintaining CO_2_ as previously described [4,29]. Our IH model cycled oxygen in the medium at 1% for 2 min, followed by 20% for 10 min, with CO_2_ maintained at 5%. Cells were also cultured under normoxia conditions (21% oxygen, 5% CO_2_) for the control group.

### 2.4. HIF1α Inhibition and Stimulation Assays

HIF1α inhibition and stimulation assays were performed using 5 × 10^5^ monocytes from HV isolated as previously described and cultured in 6-microwell plates.

For the PX478 inhibition assay, cells were treated with 30 μM of PX478 (an HIF1α inhibitor; MedKoo Biosciences Inc., Morrisville, NC, USA) [30,31] for 16 h in N or IH culture conditions [31]. For the silencing assay, the cells were transfected with 25-mM human HIF1α siRNA (s653) or a control plasmid (Thermo Fisher, Waltham, MA, USA), using an Amaxa Nucleofector (Amaxa Biosystems, Cologne, Germany). Briefly, cells were transferred to an electroporation cuvette and nucleofected, according to the manufacturer’s instructions, and then cultured for 16 h in normoxia or IH culture conditions.

For the dimethyloxalylglycine (DMOG) assay, the cells were treated with 100 μM of DMOG for 2 h in standard culture conditions.

### 2.5. Human Cutaneous Melanoma Cell Line Culture

Human cutaneous melanoma cell line C-8161 [32] was cultured in RPMI 1640 medium containing 2-mM glutamine, 50 U/mL of penicillin (Gibco, Minneapolis, MN, USA), 100 mg/mL of streptomycin (BD Biosciences, Bedford, MA, USA), and 10% fetal bovine serum (Gibco, Minneapolis, MN, USA), supplemented with 1× MITO + serum extender (Corning Inc., Lowell, MA, USA). Cells were cultured with plasma from randomized patients with OSA at a concentration of 10% or with rPSPC1 (2500 pg/mL), and/or rTGFβ (50 pg/mL), and/or αPSPC1 (1 μg/mL) either under normoxia or IH conditions. IH and normoxia conditions were the same as those used for the IH model.

### 2.6. mRNA Isolation and Quantification

Monocytes and melanoma cells were harvested and washed with phosphate-buffered saline. RNA was then isolated using the High Pure RNA Isolation Kit (Roche Diagnostics, Basel, Switzerland). Complementary DNA (cDNA) was obtained by reverse transcription of 1 μg of RNA using the High-Capacity cDNA Reverse Transcription Kit (Applied Biosystems, Waltham, MA, USA). mRNA quantification was assessed by real-time quantitative polymerase chain reaction (PCR) using the CFX96 Touch Real-Time PCR Detection System (Bio-Rad Laboratories, Hercules, CA, USA) and QuantiMix Easy SYG kit (Biotools, Madrid, Spain) with specific primers (Supplemental Information Table S1). We employed 18S as a housekeeping gene and used a 6-point standard curve to estimate the cDNA copy number of each gene of interest. All primers were synthesized, desalted, and purified by Eurofins Scientific SE (Luxembourg).

### 2.7. Flow Cytometry

The monocytes were stained after culture with antibodies against CD14 (BD Bioscience, Grenoble, France) and PSPC1 (Santa Cruz Biotechnology, Dallas, TX, USA). For the intracellular staining, the cells were treated following a standard protocol using the Transcription Factor Buffer Set (BD Biosciences). After staining for 30 min at 4 °C in the dark, the cells were acquired using a BD FACS Calibur flow cytometry from BD Biosciences (Bedford, MA, USA), and the collected data were analyzed using FlowJo vX.0.7 software (FlowJo, LLC, Ashland, OR, USA). Appropriate isotype controls were used for each experiment.

### 2.8. Determination of Plasma Levels of Soluble Proteins

Fasting venous blood samples were drawn between 10:00 and 12:00. The blood samples were centrifuged to separate the plasma, and all specimens were immediately aliquoted, frozen, and stored at −80 °C. PSPC1 (MBS9324493 from MyBioSource, San Diego, CA, USA), MMP2 (KHC3081 from Invitrogen) and TGFβ1 (LEGEND MAX™ Free Active TGF-β1 ELISA Kit 437707 from BioLegend Inc., San Diego, CA, USA), SMAD4 (CSB-E12749 from Cusabio, Houston, TX, USA), SMAD3 (EH2148 from Fine Test, Wuhan, China), MMP9 (BMS2016-2 from Invitrogen, Vienna, Austria), and GSDMD (MBS2705515 from MyBioSource, San Diego, CA, USA) were assayed using human enzyme-linked immunosorbent assay (ELISA). We followed the standard manufacturer protocol in each case. Measurements for plasma samples were obtained in duplicate. The detection limits for the assays were 31.2 pg/mL for PSPC1, 0.15 ng/mL for MMP2, 2.3 pg/mL for TGFβ, 6.25 pg/mL for SMAD4, 0.375 ng/mL for SMAD3, <1 ng/mL for MMP9, and 31.25 pg/mL for GSDMD. The intraassay and interassay variations were <20% in the various assays.

### 2.9. Western Blotting

Total cell protein extracts were obtained with RIPA buffer supplemented with a protease kit (Cat. 89900 and 78420, respectively; Thermo Scientific) from monocytes of the patients with OSA and HV. The monocytes were isolated as previously detailed. We mixed 20 μg of protein from each sample with 5× sample buffer (60-mM Tris-HCl, pH 6.8, 2% sodium dodecyl sulfate [SDS], 10% glycerol, 5% mercaptoethanol, 0.01% bromophenol blue) and subjected to Western immunoblotting. Briefly, samples were loaded onto 10% sodium dodecyl sulfate-polyacrylamide electrophoresis gel, transferred to polyvinylidene fluoride filters, and blotted against different proteins using specific antibodies for HIF1α (BD610959, BD Biosciences) and β-actin (4970, Cell Signaling Technology, Danvers, MA, USA), followed by a horseradish peroxidase-conjugated secondary immunoglobulin G (IgG) antibody (04-6020, Cell Signaling Technology). Antibody binding was detected by ECL Western Blotting Detection Reagent (Amersham-Pharmacia-Biotech, Amersham, UK).

### 2.10. Cleavage of PSPC1 by MMP2

Recombinant human MMP2 (R&D Systems) and PSPC1 were dissolved in 20-mM Tris buffer containing 2-mM CaCl_2_ and 1-mM MgCl_2_ (pH 7.3), at a concentration of 10 µg/100 L and 100 µg/100 µL, respectively. MMP2 was then added to the PSPC1 to a molar ratio of 1:10 and incubated at 37 °C for 2 h. We then added 8 µL of 1.0-M EDTA to the aliquots, which were mixed with 5× sample buffer (60-mM Tris-HCl, pH 6.8, 2% SDS, 10% glycerol, 5% mercaptoethanol, 0.01% bromophenol blue) and subjected to Western immunoblotting. Briefly, each sample was separated by SDS-polyacrylamide gel electrophoresis), and blotted onto iBlot Gel Transfer Stacks (Invitrogen). These nitrocellulose membranes were then probed with anti-PSPC1 mAb (Santa Cruz Biotechnology, Dallas, TX, USA), followed by a horseradish peroxidase-conjugated secondary IgG antibody (Cell Signaling Technology). Antibody binding was detected by ECL (Amersham-Pharmacia-Biotech).

### 2.11. PSPC1 Cleavage by MMP2 on the Monocyte Surface

Monocytes from HV were obtained and cultured as previously mentioned. After 24 h under IH or normoxia conditions, monocytes were treated with increasing concentrations of rMMP2 (2.5, 5, 10, 20, and 40 ng/mL) for 3 h. The cells were then harvested and permeabilized (for PSPC1 intracellular analysis) or not (for surface PSPC1 protein analysis) using the Transcription Factor Buffer Set (BD Biosciences). Appropriate isotype controls were used for each experiment. Lastly, the cells were stained with CD14 and PSPC1 antibodies for flow cytometry.

### 2.12. Migration Assays

To perform the wound healing assays, C-8161 cells were grown to confluence (>90%) in 24-well dishes. A small area was then disrupted by scratching the monolayer with a 200-µL plastic pipette tip. Cells were inspected microscopically at 0 and 36 h. The remaining wound area was calculated using ImageJ software (National Institutes of Health). The assay was performed using a wound-healing assay (0 h and 36 h) with TGFβ (50 pg/mL), with or without rPSPC1 (2.5 ng/mL) under normoxia and intermittent hypoxia conditions.

### 2.13. Statistical Analyses

Comparisons were performed using an unpaired *t*-test with or without Welch’s correction or two-way analysis of variance (ANOVA) depending on the variable characteristics and data distribution. Correlations were assessed with a Pearson or Spearman correlation test depending on the data distribution. The differences were considered significant at *p* < 0.05, and the analyses were conducted using Prism 8.0 software (Graph Pad, San Diego, CA, USA) and the Statistical Package for the Social Sciences 26.0 (SPSS Inc., Chicago, IL, USA).

## 3. Results

### 3.1. PSPC1 Expression in OSA Monocytes

The characteristics of the patients with OSA and HV are shown in Table 1.

**Figure 1:**
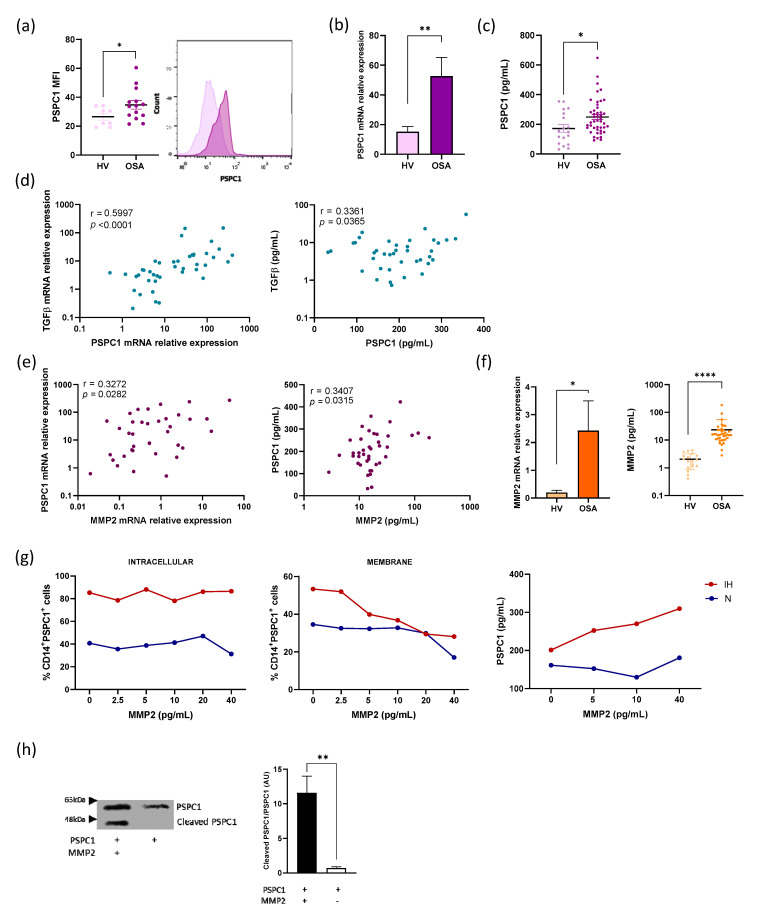
PSPC1 expression and its cleavage in patients with OSA. (**a**) The mean fluorescence intensity (MFI) of the monocyte PSPC1 intracellular expression (as determined by flow cytometry) in HV (*n* = 8) and the patients with severe OSA (*n* = 15) are shown. The PSPC1 distribution (**left** panel) and PSPC1 histogram (**right** panel) are shown; (**b**) The monocytes PSPC1 mRNA expression (estimated by qPCR) in the monocytes from HV (*n* = 23) and the patients with severe OSA (*n* = 50) are shown; (**c**) The PSPC1 plasma protein was quantified using ELISA (*n* = 18 HV and *n* = 45 patients with severe OSA). The intergroup differences in PSPC1 expression were assessed using an unpaired *t*-test with Welch’s test correction. Error bars: Standard error of the mean (SEM). * *p* < 0.05, ** *p* < 0.01 when compared with HV; (**d**) Correlation between PSPC1 and TGFβ mRNA expression in the monocytes from the patients with severe OSA (*n* = 45) (**left** panel) and the correlation between PSPC1 and TGFβ protein concentration in the plasma of the patients with severe OSA (*n* = 40) (**right** panel). The patients were randomly selected. Spearman correlation coefficients (r) and *p*-values are shown; (**e**) Correlation between MMP2 and PSPC1 mRNA expression in the monocytes from the patients with severe OSA (*n* = 45) (**left** panel) and the correlation between MMP2 and PSPC1 protein concentration in the plasma of the patients with severe OSA (*n* = 40) (**right** panel). Pearson’s correlation coefficients (r) and *p*-values are shown; (**f**) MMP2 mRNA expression analysis by qPCR in the monocytes from HV (*n* = 18) and the patients with severe OSA (*n* = 45) (**left** panel). The MMP2 protein was quantified using ELISA (*n* = 20 HV, and *n* = 40 patients with OSA) (**right** panel). The groups were compared using an unpaired *t*-test with Welch’s test correction. Error bars: SEM. * *p* < 0.05, **** *p* < 0.0001 as compared with HV; (**g**) The PSPC1 expression by flow cytometry analysis is shown. Monocytes from HV (*n* = 3) were cultured under normoxia or IH for 24 h and then treated with MMP2 for 3 h (**left** and middle panels). Supernatants were collected to evaluate PSPC1 protein concentrations using ELISA (**right** panel). Mean values are shown. Spearman coefficients (r) and *p*-values (*p*) were calculated for the **left** panel N (r = 0.3143, *p* = 0.5639) and IH (r = −0.02, *p* = >0.99) and **right** panel N (r = −0.8286, *p* = 0.0583) and IH (r = −1.00, *p* = 0.0028); (**h**) Western blot analysis of enzymatic digestion of PSPC1 with or without MMP2 (molar ratio of 1:10) is shown (**left** panel). The expected size of 59 kDa (uncleaved) and an additional band of approximately 43 kDa (cleaved) were observed. Three different experiments were performed with similar findings (**right** panel). The comparison was performed by unpaired *t*-test; ** *p* < 0.01.

Our initial approach was to explore PSPC1 expression in monocytes by flow cytometry. Our data showed a significant increase in intracellular PSPC1 expression in the monocytes from the patients with OSA compared with those from HV (*p* = 0.0393) (Figure 1a). PSPC1 mRNA expression was also consistently increased in the OSA monocytes (*p* = 0.0063) (Figure 1b). The OSA samples also displayed higher TGFβ, SMAD3 and SMAD4 mRNA expression than those from HV (*p* = 0.0044, *p* = 0.0457 and *p* = 0.0019, respectively) (Figure S1a).

We identified high plasma PSPC1 protein levels in the patients with OSA compared with HV (*p* = 0.017) (Figure 1c) and observed a similar pattern with TGFβ, SMAD3, and SMAD4 (*p* = 0.0193, *p* = 0.0008 and *p* < 0.0001, respectively) (Figure S1b). In the patients with OSA, we found a strong correlation between PSPC1 mRNA levels and TGFβ mRNA expression, a correlation that was maintained with PSPC1 and TGFβ plasma proteins (Figure 1d). All these data suggest an association between elevated PSPC1 levels and TGFβ pathway activity in patients with OSA.

### 3.2. Cleavage of PSPC1 by MMP2

The function of PSPC1 inside the cell has been well described; however, its presence in the extracellular space has not yet been reported (to our knowledge). To explore the possible mechanisms involved in PSPC1 protein release into the plasma from the monocytes of patients with OSA, we first examined the correlation between PSPC1 levels and gasdermin D, which is involved in the pyroptosis pathway [33,34]. Gasdermin D expression showed no association with PSPC1 or TGFβ in either mRNA expression or protein concentration (Figure S2a,b). We then explored the association with metalloproteinases, such as MMP9 and MMP2. Whereas MMP9 was not associated with PSPC1 (Figure S2c,d), we did find an association between MMP2 and PSPC1 mRNA expression (r = 0.3272 and *p* = 0.0282), and this correlation was maintained at protein levels (r = 0.3407 and *p* = 0.03915) (Figure 1e). We also found a positive correlation between MMP2 and TGFβ protein expression, although mRNA expression showed no statistical significance (Figure S2e). Furthermore, MMP2 levels were higher in the patients with OSA than in HV for both mRNA expression and protein concentration (*p* = 0.0445 and *p* < 0.0001, respectively) (Figure 1f). We, therefore, evaluated the functional role of MMP2 in the release of PSPC1 from monocytes. We used an in vitro model with monocytes from HV in the presence of rMMP2 protein at various doses to evaluate the PSPC1 on the surface and the intracellular expression under normoxia and IH conditions. Our data suggest that PSPC1 expression significantly decreases on the cell surface along with rMMP2 concentration only under IH conditions. However, PSPC1 intracellular expression remains at the same level with rMMP2 increasing concentration. Moreover, we found that the significant increase in PSPC1 protein levels in the supernatant of monocytes treated with rMMP2 only occurs in IH (Figure 1g). Furthermore, these data also showed that PSPC1 expression increased under IH.

To analyze PSPC1 cleavage by MMP2, we performed an enzymatic digestion test with PSPC1 as a target protein and rMMP2 as an enzyme. We then evaluated PSPC1 cleavage by western blot using an anti-PSPC1 antibody. Our results indicated that MMP2 is involved in PSPC1 cleavage (Figure 1h).

### 3.3. PSPC1 Expression Is Mediated by Intermittent Hypoxemia

**Figure 2:**
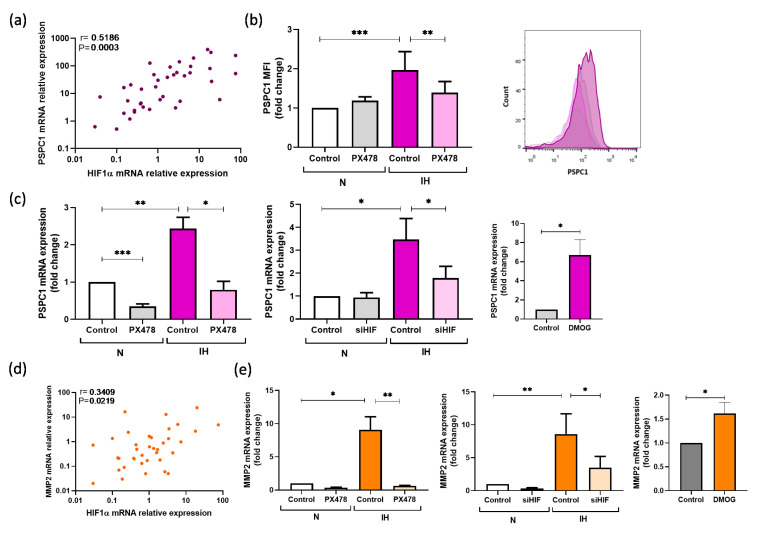
HIF1α is associated with PSPC1. (**a**) Correlation between PSPC1 and HIF1α mRNA expression in monocytes in patients with OSA (*n* = 40). Pearson correlation coefficients (r) and *p*-values are shown; (**b**) Monocytes from HV (*n* = 7) were treated with 30 μM of PX478 and/or exposed to IH for 16 h. Paired control samples were incubated under normoxia conditions and without PX478. The distribution estimation of MFI of the PSPC1 intracellular expression (as determined by flow cytometry, **left** panel) and the PSPC1 histogram (**right** panel) are shown; (**c**) PSPC1 mRNA expression estimated by qPCR in the monocytes from HV (*n* = 7) treated with a specific HIF1α inhibitor (30 μM of PX478) or not and exposed to IH or normoxia conditions for 16 h (**left** panel), monocytes from HV (*n* = 3) treated with or without siHIF1α and exposed to IH or normoxia conditions for 16 h (middle panel), monocytes from HV (*n* = 3) treated or not with DMOG for 2 h (**right** panel); (**d**) Correlation between MMP2 and HIF1α mRNA expression in the monocytes from the patients with severe OSA (*n* = 40). Spearman’s correlation coefficients (r) and *p*-values are shown; (**e**) MMP2 mRNA expression estimated by qPCR in the monocytes from HV (*n* = 7) treated with a specific HIF1α inhibitor (30 μM of PX478) or not and exposed to IH or normoxia conditions for 16 h (**left** panel), monocytes from HV (*n* = 3) treated with or without siHIF1α and exposed to IH or normoxia conditions for 16 h (middle panel), monocytes from HV (*n* = 3) treated or not with DMOG for 2 h (**right** panel). The groups were compared with a Two-way ANOVA or paired *t*-test. Error bars: SEM. * *p* < 0.05; ** *p* < 0.01, *** *p* < 0.001, compared with the untreated cells. Spearman’s correlation coefficients (r) and *p*-values are shown.

The hypoxic status of the patients with OSA has been corroborated by the higher HIF1α expression compared with HV (Figure S3a). In OSA monocytes, HIF1α mRNA expression was significantly related to both PSPC1 (r = 0.5161, *p* = 0.0003) (Figure 2a) and TGFβ mRNA expression (r = 0.3531, *p* = 0.0173) (Figure S3b). To verify the contribution of HIF1α to hypoxia-mediated upregulation of PSPC1, we exposed monocytes from HV to IH conditions in combination with agent PX478 (S-2-amino-3-[4V-N,N,-bis(2-chloroethyl) amino]-phenyl propionic acid N-oxide dihydrochloride), which suppresses constitutive and hypoxia-induced levels of HIF1α (Figure S3c). The increase in PSPC1 intracellular protein and mRNA expression in monocytes under IH disappeared when the cells were treated with PX478 (Figure 2b,c, respectively). Furthermore, we performed silencing and enhancing assays targeting HIF1α (Figure S3c), to corroborate that PSPC1 is modulated by HIF1α (Figure 2c).

Moreover, TGFβ mRNA expression also increased under IH conditions compared with normoxia, and it decreased under PX478 or siHIF treatment. In this line, the HIF1α enhancing by DMOG also showed an increased TGFβ mRNA expression (Figure S3d).

Following the same strategy, we also evaluated the IH effect on MMP2 expression, finding an association between HIF1α and MMP2 mRNA expression in the patients with OSA (r = 0.3409, *p* = 0.0219) (Figure 2d). Furthermore, we evaluated the role of HIF1α in MMP2 using the HIF1α modulation in vitro assays, which showed that IH and DMOG treatment increases MMP2 mRNA levels, while HIF1α suppression reduces MMP2 mRNA expression (Figure 2e).

To assess the possible mechanisms underlying HIF1α-induced PSPC1 overexpression in patients with OSA, we searched for hypoxia response elements (HRE) motifs on the PSPC1 promoter using the TRANSFAC^®^ database. We also evaluated the HRE motifs on the TGFβ and MMP2 promoters. We found four possible binding sites for HIF1α in the PSPC1 promoter. Moreover, we found one and two HREs in the promoter of TGFβ and MMP2 genes, respectively. Lastly, many of these HREs were located in highly conserved DNase hypersensitivity sites (Figure S3e). The exact position of the motifs, as well as the sequences and the values for matrix and core similarity, are provided in Table S2.

### 3.4. IH Increases the Effect of PSPC1 on the TGFβ Pathway in Tumor Cells

**Figure 3:**
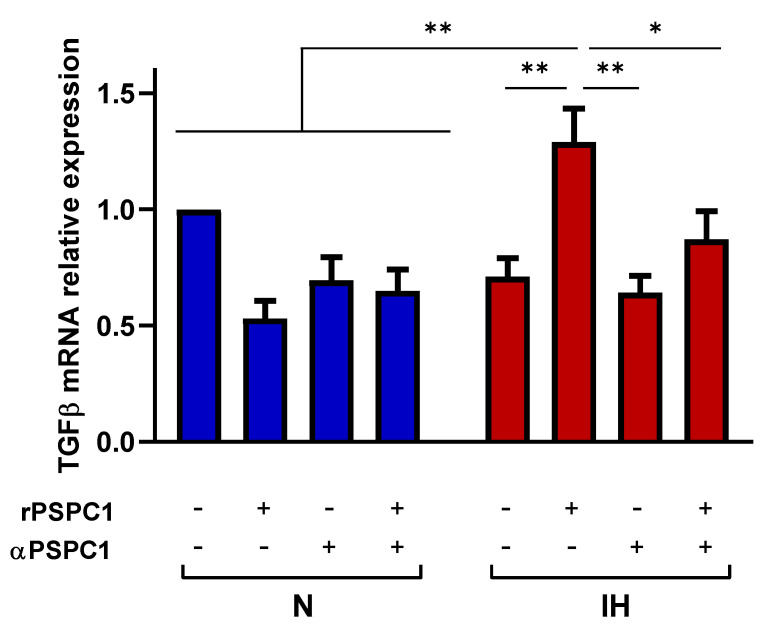
Hypoxemia collaborates with PSPC1 to modulate TGFβ expression. The melanoma cell line (C-8161) was cultured under N and IH conditions with rPSPC1 protein (2.5 ng/mL) with or without α-PSPC1 (1 ug/mL) for 16 h. The melanoma cells were then harvested to analyze TGFβ mRNA expression by qPCR. The groups were compared using two-way ANOVA. Error bars: SEM. * *p* < 0.01, ** *p* < 0.001 are shown.

To evaluate the specific effect of PSPC1 in combination with IH on TGFβ mRNA expression capacity, we used a functional in vitro model with a human melanoma cell line (C-8161) under normoxia and IH conditions. Melanoma cells were cultured with or without PSPC1 protein (rPSPC1) in the presence or absence of a human PSPC1 antibody (α-PSPC1). The data showed that rPSPC1 increases TGFβ mRNA expression only under IH conditions, while PSPC1 blockage decreases TGFβ expression (Figure 3).

### 3.5. PSPC1 Effect on the Expression of EMT and CSC Transcription Factors in Melanoma Cells under IH Conditions

**Figure 4:**
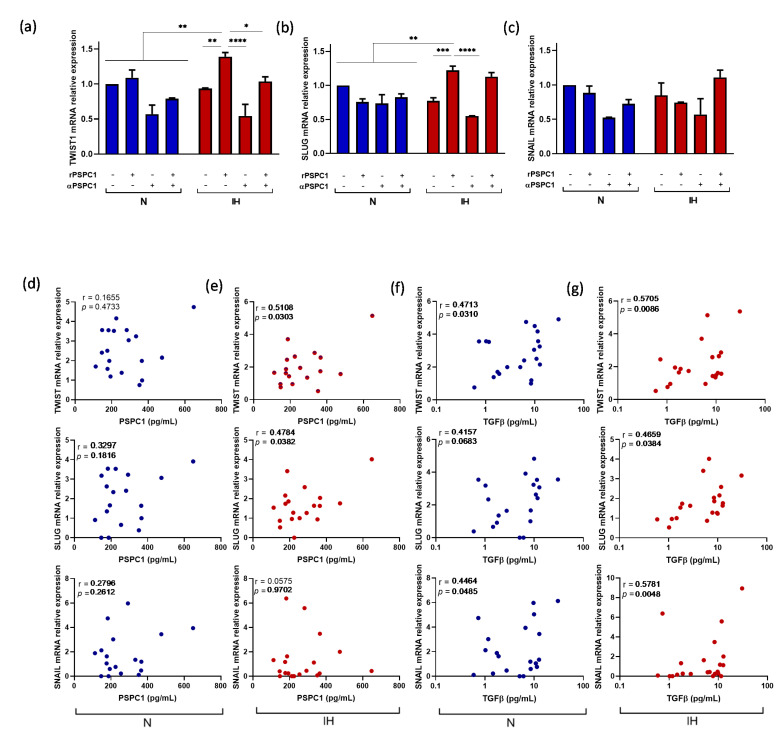
Effect of PSPC1 on EMT-TFs expression in a melanoma cell line. The melanoma cell line (C-8161) was cultured under N and IH conditions with rPSPC1 protein (2.5 ng/mL) with or without α-PSPC1 (1 ug/mL) for 16 h. The melanoma cells were then harvested to analyze TWIST (**a**), SLUG (**b**), and SNAIL (**c**) mRNA expression by qPCR. The groups were compared using a two-way ANOVA. Error bars: SEM. * *p* < 0.05, ** *p* < 0.01, *** *p* < 0.001, and **** *p* < 0.0001. The melanoma cell line (C-8161) was cultured under IH and N conditions with plasma from randomly selected patients with severe OSA (10% concentration) for 16 h. The melanoma cells were then harvested to analyze TWIST, SLUG, and SNAIL mRNA expression by qPCR analysis. The correlation between PSPC1 protein concentrations from OSA plasma supplemented to the cell culture with mRNA expression of EMT-TFs in N (**d**) and IH (**e**) conditions are shown. The correlation between TGFβ protein concentrations from OSA plasma supplemented to the cell culture and mRNA expression of EMT-TFs in N (**f**) and IH (**g**) conditions are shown. Pearson’s correlation coefficients (r) and *p*-values are shown.

Using the functional model of a melanoma cell line under normoxia and IH, with PSPC1 recombinant protein and human PSPC1 antibody, our data indicated a significant PSPC1 effect on EMT activation through TWIST and SLUG only under IH conditions (Figure 4a,b). However, no effect was found on SNAIL mRNA expression in normoxia or IH (Figure 4c). Moreover, the PSPC1 effect on CSC activation data showed only a relevant effect on SOX2 mRNA expression under IH. However, NANOG and OCT3/4 did not reach statistically significant differences (Figure S4a–c). These results suggest that PSPC1 might play a role in EMT under IH conditions.

To elucidate the effects of circulating PSPC1 protein, we performed an in vitro assay using plasma from patients with OSA in the melanoma cells under normoxia or IH conditions. We found a positive correlation between PSPC1 plasma concentration and TGFβ mRNA expression only under IH (r = 0.5079, *p* = 0.0314). Similarly, the PSPC1 concentration in the IH cultures was significantly associated with increased TWIST (r = 0.5108, *p* = 0.0303) and SLUG (r = 0.4784, *p* = 0.0382) mRNA expression, while no correlation was found under normoxia conditions (Figure 4d,e). The correlations found in normoxia between TGFβ concentrations of OSA plasma used to supplement the cultures and TWIST, SLUG, and SNAIL mRNA expression (r = 0.4713, *p* = 0.0310; r = 0.4157; *p* = 0.0680; and r = 0.4464, *p* = 0.0485, respectively) were improved under IH conditions (r = 0.5705, *p* = 0.0086; r = 0.4659, *p* = 0.0384; and r = 0.5781, *p* = 0.0048, respectively) (Figure 4f,g).

We also evaluated the in vitro capability of plasma concentrations of PSPC1 and TGFβ protein to induce CSC-TF expression in melanoma cells, finding that SOX2 might be regulated by both proteins under IH conditions (Figure S4d–g). However, no effects on NANOG or OCT3/4 mRNA expression were found (Figure S4d–g).

Lastly, we studied the correlation between PSPC1 and TGFβ mRNA expression on the melanoma cell line with EMT-TF and CSC-TF transcription factor expression under either IH or normoxia conditions. Our data confirmed that IH significantly increases the correlation compared with normoxia (Table S3).

### 3.6. PSPC1 and TGFβ Have a Synergistic Effect on EMT-TF and CSC-TF Expression under IH

**Figure 5:**
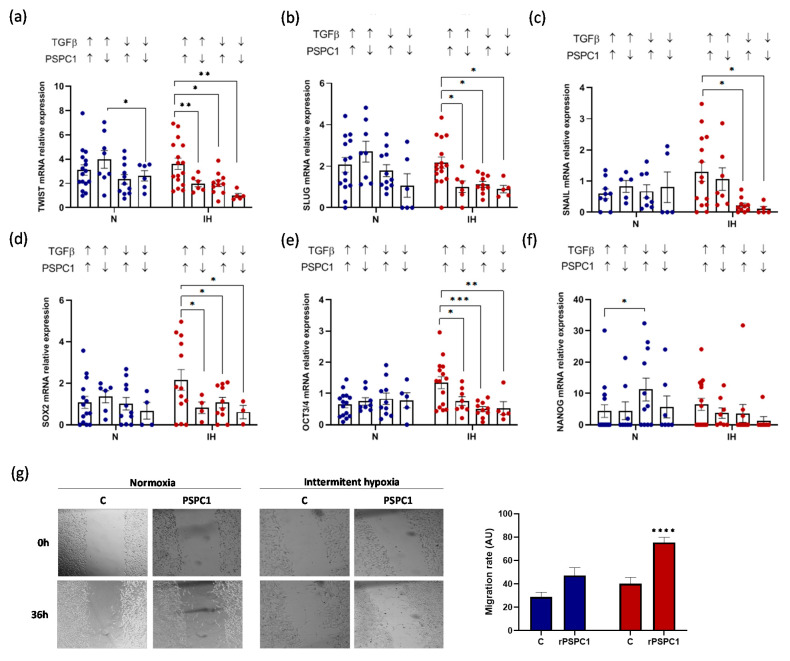
The synergistic effect of PSPC1 and TGFβ enhances the expression of EMT-TFs. The melanoma cell line was cultured under normoxia and IH conditions with plasma from patients with OSA (10% concentration) for 16 h. OSA plasma was selected according to PSPC1 and TGFβ protein concentrations. We established four groups: PSPC1-high (200–2000 pg/mL) with TGFβ-high (5–100 pg/mL) as group 1, PSPC1-low (30–200 pg/mL) with TGFβ-high (5–100 pg/mL) as group 2, PSPC1-high (200–2000 pg/mL) with TGFβ-low (0–5 pg/mL) as group 3, and PSPC1-low (30–200 pg/mL) with TGFβ-low (0–5 pg/mL) as group 4. The melanoma cells were then harvested to analyze the mRNA expression by qPCR analysis. EMT transcription factor mRNAs were analyzed through TWIST (**a**), SLUG (**b**), and SNAIL (**c**) mRNA expression estimation by qPCR. CSC transcription factor mRNAs were analyzed through SOX2 (**d**), OCT3/4 (**e**), and NANOG (**f**) mRNA expression. The comparison between groups was performed with a two-way ANOVA. Error bars: SEM. * *p* < 0.05, ** *p* < 0.001 and *** *p* < 0.0001 compared with the treated group, respectively. (**g**) C-8161 cells were incubated with TGFβ (50 pg/mL), with or without PSPC1 (2,5 ng/mL) under normoxia and intermittent hypoxia for 36 h. Representative cell images from each group in the indicated time points are shown; (**left** panels). Quantification of wound healing is shown (**right** panels). Data were analyzed by two-way ANOVA. Results are expressed as means ± SEM (*n* = 3). **** *p* < 0.0001.

To determine a potential synergistic effect, we selected OSA plasma by PSPC1 and TGFβ protein concentrations to add to the melanoma cell culture under conditions of IH or normoxia to evaluate the EMT outcome. Our data showed that PSPC1 protein significantly increases TGFβ mRNA expression only under IH conditions, and it does so more intensely in combination with TGFβ protein (Figure S4h). Interestingly, the combination of PSPC1 and TGFβ proteins showed a synergic effect on EMT-TF (TWIST and SLUG) under IH conditions (Figure 5a,b), whereas SNAIL expression increased only upon stimulation with high TGFβ levels, regardless of PSPC1 levels (Figure 5c).

These results are consistent with those shown in Figure 4, where SNAIL mRNA levels correlate with TGFβ protein levels but not with PSPC1. PSPC1 and TGFβ also exert a synergistic effect on CSC-TF expression under IH conditions, particularly on SOX2 and OCT3/4 mRNA expression (Figure 5d–f). To support these data, we performed an in vitro assay using various concentrations of rPSPC1 protein in the presence or absence of rTGFβ protein under normoxia and IH (Figure S6). Moreover, we performed a wound-healing assay to determine the effect of PSPC1 and IH on EMT. Our data showed that rPSPC1 increased the migration capacity of melanoma cells. The migration rate significantly increased after 36 h when cells were treated with PSPC1 in combination with TGFβ under IH conditions, corroborating the effect of PSPC1 on EMT (Figure 5g).

Growing evidence have demonstrated that tumor cells exhibit hybrid epithelial-mesenchymal phenotype. Therefore, we explored the expression of epithelial marker such as E-cadherin. We found no changes in E-cadherin expression in the melanoma cells in the presence of rPSPC1 with or without αPSPC1 under IH or normoxia conditions (Figure S5a). In line with these findings, we observed no correlation between E-cadherin mRNA expression and PSPC1 or TGFβ protein content in OSA plasma, either in normoxia or IH (Figure S5b–e). Interestingly, we observed a synergic effect of TGFβ and PSPC1 as evaluated by E-cadherin mRNA expression under normoxia conditions; however, we observed no significant changes under IH conditions (Figure S5f).

Collectively, our data suggest that IH induces migration and EMT-TF and CSC-TF expression in melanoma cells stimulated with PSPC1 and TGFβ, which highlights the relevance of hypoxemia in inducing a tumor microenvironment that facilitates a synergism by means of PSPC1, switching TGFβ activation that is associated with tumor progression by EMT-TF and CSC-TF.

## 4. Discussion

Our study shows that PSPC1 is overexpressed in circulating monocytes from patients with OSA with no evidence of cancer in a HIF1α-dependent manner. Moreover, the increase in intracellular PSPC1 is accompanied by higher plasma PSPC1 levels, probably by a cut induced by MMP2. In turn, both intracellular and plasma PSPC1 levels are associated with TGFβ upregulation, with higher levels of mRNA expression and plasma protein concentrations.

Although previous evidence has shown that PSPC1 is strongly associated with TGFβ expression [25], this finding in monocytes from patients with OSA provides biological support for the increased tumor risk, cancer aggressiveness, and mortality due to cancer that has been reported in these patients. Additionally, in patients with OSA, the TGFβ levels has been implicated in the phenotypic change of monocytes/macrophages, with a switch to tumor-associated macrophages [35] and in the suppression of the maturation and cytotoxic capacity of natural killer cells [6], which seriously compromises the immunosurveillance system. In this situation, together with the IH-induced, HIF1α dependent, overexpression of the programmed death-1/programmed death-ligand 1 immune checkpoint [5], the limited ability to block cells that arise with neoplastic transformation could explain the higher incidence of tumors. Moreover, OSA causes a modulation of the microenvironment that favors cancer progression through the persistence of immunosuppression and the induction of various proangiogenic factors, such as vascular endothelial growth factor [7] and midkine [36]. However, the TGFβ induction mediated by PSPC1 suggests that OSA could also affect the intrinsic properties of eventual tumor cells promoting their aggressiveness. In fact, elevated TGFβ expression and activation of TGFβ receptor-initiated intracellular signaling in tumor microenvironments are observed to facilitate tumor metastasis in many cancer types [25].

In addition to the increased PSPC1 expression in the monocytes of patients with OSA regulated by HIF1α transcriptional pathways, the high circulating PSPC1 levels found in these patients could contribute to a paracrine effect. Furthermore, it has been reported that hypoxia, by HIF1α, induces MMP2 expression in endothelial cells, cardiac fibroblasts, macrophages, and breast cancer cells [37,38,39,40]. Hypoxia also promoted TGFβ1/Smad signaling via HIF1α [41]. This study suggests that the increased PSPC1 cleavage caused by the higher concentration of MMP2 explains how the PSPC1 protein located in the interchromatin space of the cell nucleus is released into the circulation [22]. These results suggest that there are probably other mechanisms in addition to transcriptional activation, as other authors have also reported on MMP activity [42]. Moreover, TGFβ levels correlate with MMP in a cell type-specific manner. For example, while TGFβ expression is associated with MMP9 rather than MMP2 in transformed keratinocyte cells [43], in pancreatic tumor cells there is an association with MMP2 but not with MMP9 [44].

Interestingly, MMP2 and MMP9 are largely involved with hypoxia [45,46,47], tumor progression [48,49], and immune evasion [50]. Moreover, TGFβ is activated by MMP2 through the proteolytic cleavage of LAP [51,52], and a hypoxic environment induces TGFβ signaling [53]. Along these lines, IH conditions increase TGFβ activation in the monocytes of patients with OSA, resulting in high serum levels of the activated TGFβ form [6]. This evidence suggests that hypoxemia conditions during IH are associated with enhanced MMP and TGFβ activity. Additionally, PSPC1 enhances matrix metalloproteinases by suppressing physical barriers such as the extracellular matrix, which facilitates the progression of metastasis [23].

To assess the potential functional impact of PSPC1 and TGFβ activation in patients with OSA when they develop a tumor, we used an in vitro model of melanoma cells, given that melanoma aggressiveness is clearly elevated in these patients [1,54]. This model confirms that IH potentiates the effect of PSPC1 on TGFβ and that PSPC1 has a paracrine effect on the expression of EMT transcription factors by cancer cells, both when it is used as a recombinant protein and when used in the plasma from patients with OSA without evidence of cancer. In tumor cells, PSPC1 interacts with SMAD2/3 in the nucleus through its NOPS-coil domain in a TGFβ-dependent manner. This interaction activates TGFβ autocrine signaling mediated by the various gene transcription factors of EMT and CSC to potentiate pro-metastatic reprogramming gene expression in tumor progression [24]. Complementarily, our results show that high PSPC1 levels contained in the plasma of patients with OSA maintain a paracrine effect when exposed to tumor cells, inducing the overexpression of certain EMT transcription factors such as TWIST and SLUG. In addition, the EMT is a biological process that implies heterogeneous phenotypes resulting in intermediate states involving both characteristics of mesenchymal and epithelial phenotypes [55,56,57]. In this line, we explored E-cadherin as an epithelial marker. Our results suggest that PSPC1 is capable to induce the transition to mesenchymal phenotype by diminishing E-cadherin while increasing the EMT-TF. However, our in vitro results show a discrete effect on CSC, inducing higher SOX2 expression only. It is interesting to consider that the EMT and CSC programs share features in different complex situations orchestrated and synchronized by a series of master EMT-inducing transcription factors [58,59]. There is evidence that activation of the SNAIL axis alone is insufficient for driving the conversion to a CSC phenotype. TWIST overexpression also did not expand the CSC subpopulations, while the joint action of SNAIL and TWIST might induce whole processes in progressive stages of EMT and CSC conversion [60,61]. These data might explain the reduced effect of PSPC1 on CSC-TF expression, given that PSPC1 levels contained in plasma from patients with OSA do not show the capacity to increase SNAIL expression, and consequently TWIST signaling fails to induce a strong effect on CSC.

Lastly, it is interesting to highlight the synergistic effect when tumor cells are exposed to high concentrations of PSPC1 and TGFβ in the presence of IH. This effect could be explained by the TGFβ dependency of the PSPC1-SMAD2/3 complex binding [26]. However, it should also be noted that PSPC1 can promote tumor aggressiveness in other ways. PSPC1 also facilitates the cytoplasmic translocation of protein tyrosine kinase 6 (PTK6) to become an oncogene, and β-catenin nuclear translocation to interact with PSPC1 for augmenting Wnt3a autocrine signaling and tumor progression [62]. Evidence shows that PSPC1 augments cell adhesion and motility by promoting insulin-like growth factor 1 receptor expression to stimulate downstream focal adhesion and integrin signaling pathways [63], as well as activation of EMT and CSC, in association with a higher risk of metastasis and a poorer prognosis for patients with cancer [24,64].

## 5. Conclusions

**Figure 6:**
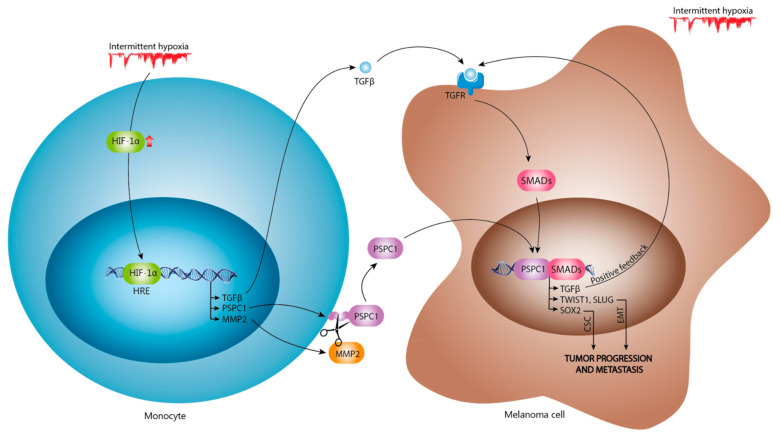
PSPC1 expression in patients with OSA increases TGFβ expression effect on EMT-TFs. Intermittent hypoxia increases the activation of HIF1α, which might bind with the promoter of genes such as PSPC1, MMP2, and TGFβ, leading to high levels of these proteins in severe OSA monocytes. The MMP2 plays a role in PSPC1 cleavage, increasing plasma PSPC1 levels. The combination of high plasma levels of TGFβ and PSPC1 increases the EMT-TF effect through TWIST and SLUG and CSC effect by SOX2 in melanoma cells.

Our data reveal that the effects of IH might contribute to increased PSPC1 levels in patients with OSA. These hypoxemic microenvironment variations increase HIF1α, which activates the transcription of PSPC1, MMP2, and TGFβ genes. PSPC1 is released from OSA monocytes into the circulation through MMP2. PSPC1 protein expression is accompanied by enhanced TGFβ, which ultimately switches from the immune surveillance role to the tumor progression function. We have corroborated our hypothesis with a melanoma cell model as a functional model to demonstrate that, under IH, the PSPC1 increases TGFβ expression and promotes EMT, through TWIST and SLUG transcription factors, as well as CSC, although only by means of SOX2 (Figure 6). Thus, we propose that PSPC1 activity essentially amplifies critical tumor progression by EMT, which might explain the high cancer aggressiveness in patients with OSA.

## Figures and Tables

**Table 1:** General characteristics of the study participants *.

Variables	Patients with Severe OSA (*n* = 50)	Healthy Volunteers (*n* = 20)	*p*
Male sex, *n* (%)	36 (72)	15 (75)	0.525
Age, years	59 ± 12	56 ± 8	0.418
Weight, kg	92 ± 22	84 ± 8	0.332
Body mass index, kg/m^2^	32.6 ± 6.9	30.5 ± 1.9	0.370
Neck circumference, cm	42 ± 9	41 ± 8	0.413
Smoking habit, *n* (%)			0.231
Current smoker	16 (32)	6 (30)	
Former smoker	12 (24)	4 (20)	
Never smoker	22 (44)	10 (50)	
Epworth Sleepiness Scale	8.7 ± 4.2	2.0 ± 0.8	<0.001
AHI, events/h	53.6 ± 16.9	2.7 ± 1.2	<0.001
Oxygen desaturation index, events/h	51.0 ± 17.1	1.9 ± 1.1	<0.001
Recording time with SpO_2_ < 90%, %	31.7 ± 28.9	2.3 ± 2.1	<0.001
Mean nocturnal SpO_2_, %	90.6 ± 3.2	93.3 ± 1.5	0.003
Lowest nocturnal SpO_2_, %	76.3 ± 8.9	90.2 ± 1.3	<0.001

* Data are expressed as mean ± SD or number (percentage). Abbreviations: AHI, apnea-hypopnea index; SpO_2_, oxyhemoglobin saturation; OSA, obstructive sleep apnea. Comparisons between groups were performed by Student’s *t*-test or the chi-squared test.

## Data Availability

Data are available upon reasonable request.

## Supplementary Materials

The following are available online at https://www.mdpi.com/article/10.3390/cancers13153888/s1, Table S1: qPCR Primer sequences are shown, Table S2: Position, Matrix Score and Sequence from the EBOX and HRE motifs, Table S3: Correlation of mRNA expression of PSPC1, TGFβ and markers of EMT and CSC in patients with OSA, Figure S1: TGFβ, SMAD3 and SMAD4 expression in monocytes from severe patients with OSA, Figure S2: The PSPC1 and TGFβ expression correlations with GSDMD and MMP9, Figure S3: Hypoxemia is associated with TGFβ and MMP2 expression in OSA monocytes, Figure S4: PSPC1 expression in patients with OSA increases the effect on CSC, Figure S5: PSPC1 and TGFβ expression in OSA patients effect on E-cadherin expression, Figure S6: rPSPC1 effect on EMT.
