# Supplementary material for: Intermittent Hypoxia Mediates Paraspeckle Protein-1 Upregulation in Sleep Apnea
Source: Cancers (Basel). 2021 Aug 2;13(15):3888. doi: 10.3390/cancers13153888 (PMC8345391; doi:10.3390/cancers13153888)
Supplement: Supplementary file 1 [file cancers-13-03888-s001.zip › cancers-1294958-supplementary.pdf]

Supplemental Information

Title: **Intermittent hypoxia mediates paraspeckle protein-1 upregulation in sleep apnea**

Authors: Elena Díaz-García, Sara García-Tovar, Raquel Casitas, Ana Jaureguizar, Ester Zamarrón, Begoña Sánchez-Sánchez, Ana Sastre-Perona, Eduardo López-Collazo, Francisco García-Río, Carolina Cubillos-Zapata

Table S1. qPCR Primer sequences are shown.

PSPC1	Forward primer	GACGCTTGGAAGAACTCAGA
	Reverse primer	TTGGAGGAGGACCTTGGTTAC
TGFβ	Forward primer	CTAATGGTGGAAACCCACAACG
	Reverse primer	TATCGCCAGGAATTGTTGCTG
SMAD3	Forward primer	AACGGCCAGGAGGAGAAATG
	Reverse primer	ATCCAGGGACCTGGGGATG
SMAD4	Forward primer	TGCATTCCAGCCTCCCATTT
	Reverse primer	CTCTCCTACCTGAACGTCCATT
GSDMD	Forward primer	GTGTGTCAACCTGTCTATCAAGG
	Reverse primer	CATGGCATCGTAGAAGTGGAAG
MMP2	Forward primer	GATACCCCTTTGACGGTAAGGA
	Reverse primer	CCTTCTCCCAAGGTCCATAGC
MMP9	Forward primer	TTCCAAACCTTTGAGGGCGA
	Reverse primer	CAAAGGCGTCGTCAATCACC
HIF1a	Forward primer	TTCCAGTTACGTTCCTTCGATCA
	Reverse primer	TTTGAGGACTTGCGCTTTCA
TWIST1	Forward primer	GGAGTCCGCAGTCTTACGAG
	Reverse primer	TCTGGAGGACCTGGTAGAGG
SLUG	Forward primer	GGGGAGAAGCCTTTTTCTTG
	Reverse primer	TCCTCATGTTTGTGCAGGAG
SNAIL	Forward primer	CCAGGCTGAGGTATTCCTTG
	Reverse primer	CCTCCCTGTCAGATGAGGAC
NANOG	Forward primer	TGAACCTCAGCTACAAACAGGTC
	Reverse primer	AACTGCATCCAGGACTGCAGAG
OCT3/4	Forward primer	CTTGCTGGAGAAGTGGGTGGAA
	Reverse primer	CTGGAGTGTGGGTTTCGGGCA
SOX2	Forward primer	AGAACCCCAAGATGCACAAC
	Reverse primer	CGGGGCCGGTATTTATAATC
E-cadherin	Forward primer	TGCCCAGAAAATGAAAAAGG
	Reverse primer	GTGTATGTGGCAATGCGTTC
18S	Forward primer	CGGCGACGACCCATTCGAAC
	Reverse primer	GAATCGAACCCTGATTCCCCGTC

Table S2. Position, Matrix Score and Sequence from the EBOX and HRE motifs*

	Motif	Core Similarity	Matrix Similarity	Start Position	End Position	Sequence
PSPC1	HRE	0.997	0.985	19783083	19783090	CGCGTGCA
	HRE	0.997	0.970	19785717	19785724	GGCACGCT
	HRE	0.825	0.806	19787950	19787957	AGTGTGCC
TGFβ	HRE	0.828	0.791	41353977	41353990	AAGCGCACGGGGCG
	HRE	0.793	0.773	41362572	41362585	TTCTAAGTGCTGTT
MMP2	HRE	0.835	0.803	55470847	55470860	CCCTGCGTGCCACC
	HRE	0.835	0.825	55480545	55480558	GTGAGCGTGCGCGC

* These data were obtained TRANSFAC® database

Table S3. Correlation of mRNA expression of PSPC1, TGFβ and markers of EMT and CSC in OSA patients

**Intermittent hypoxia**							
	**TGFβ**	**TWIST**	**SNAIL**	**SLUG**	**OCT3/4**	**SOX2**	**NANO G**
**PSPC 1**	r=0.761 p<0.001	r=0.509 p=0.016	r=0.561 p=0.015	r=0.539 p=0.010	r=0.491 p=0.028	r=0.030 p=0.916	r=0.777 p<0.001
**TGFβ**		r=0.577 p=0.005	r=0.693 p=0.001	r=0.544 p=0.009	r=0.399 p=0.082	r=0.237 p=0.395	r=0.696 p<0.001
**Normoxia**							
	**TGFβ**	**TWIST**	**SNAIL**	**SLUG**	**OCT3/4**	**SOX2**	**NANO G**
**PSPC 1**	r=0.285 p=0.199	r=0.251 p=0.261	r=0.246 p=0.296	r=0.225 p=0.315	r=0.143 p=0.536	r=0.240 p=0.307	r=0.223 p=0.320
**TGFβ**		r=0.198 p=0.376	r=0.659 p=0.002	r=0.136 p=0.545	r=0.231 p=0.313	r=0.334 p=0.150	r=0.566 p=0.006

Correlation values between PSPC1, TGFβ and SMAD3 mRNA with EMT-TF or CSC-TF mRNA Melanoma cell line was cultured under IH and normoxia conditions with plasma from OSA patients (10% concentration) during 16h. Pearson's correlation coefficient (r) and p-values from correlations between PSPC1, TGFβ or SMAD3 mRNA expression and the different EMT-TFs and CSC-TFs mRNA expression are shown.

FIGURES

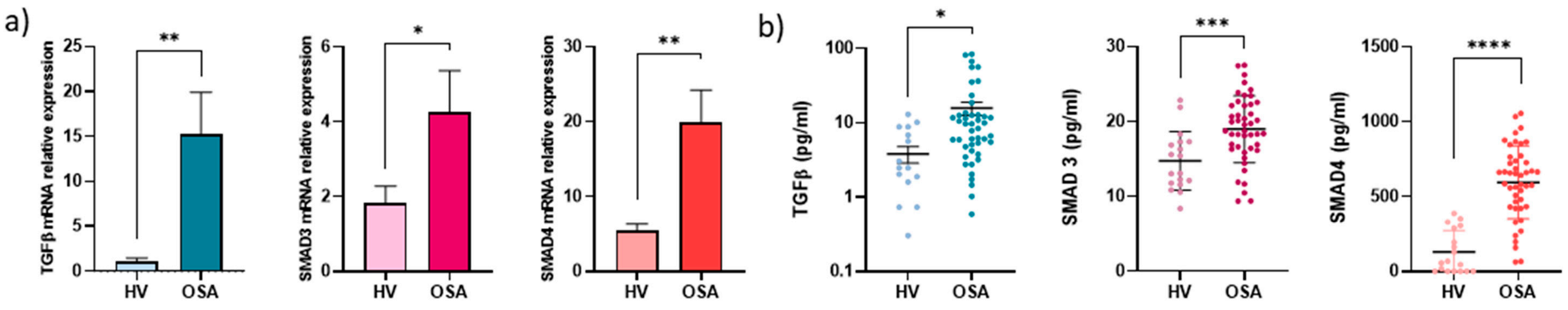

Figure S1. TGFβ, SMAD3 and SMAD4 expression in monocytes from severe OSA patients.

a) The TGFβ, SMAD3 and SMAD4 mRNA expression analysis by qPCR from healthy volunteers (HV) (n=18) and severe OSA patients (n=45) are shown. b) The TGFβ, SMAD3 and SMAD4 protein expression analysis by ELISA from HV (n=18) and severe OSA (n=45) plasma are shown. Comparisons between groups were performed by paired t-test. Error bars: SEM.*:p<0.05, **:p<0.01, ***:p<0.001; ****:p<0.0001.

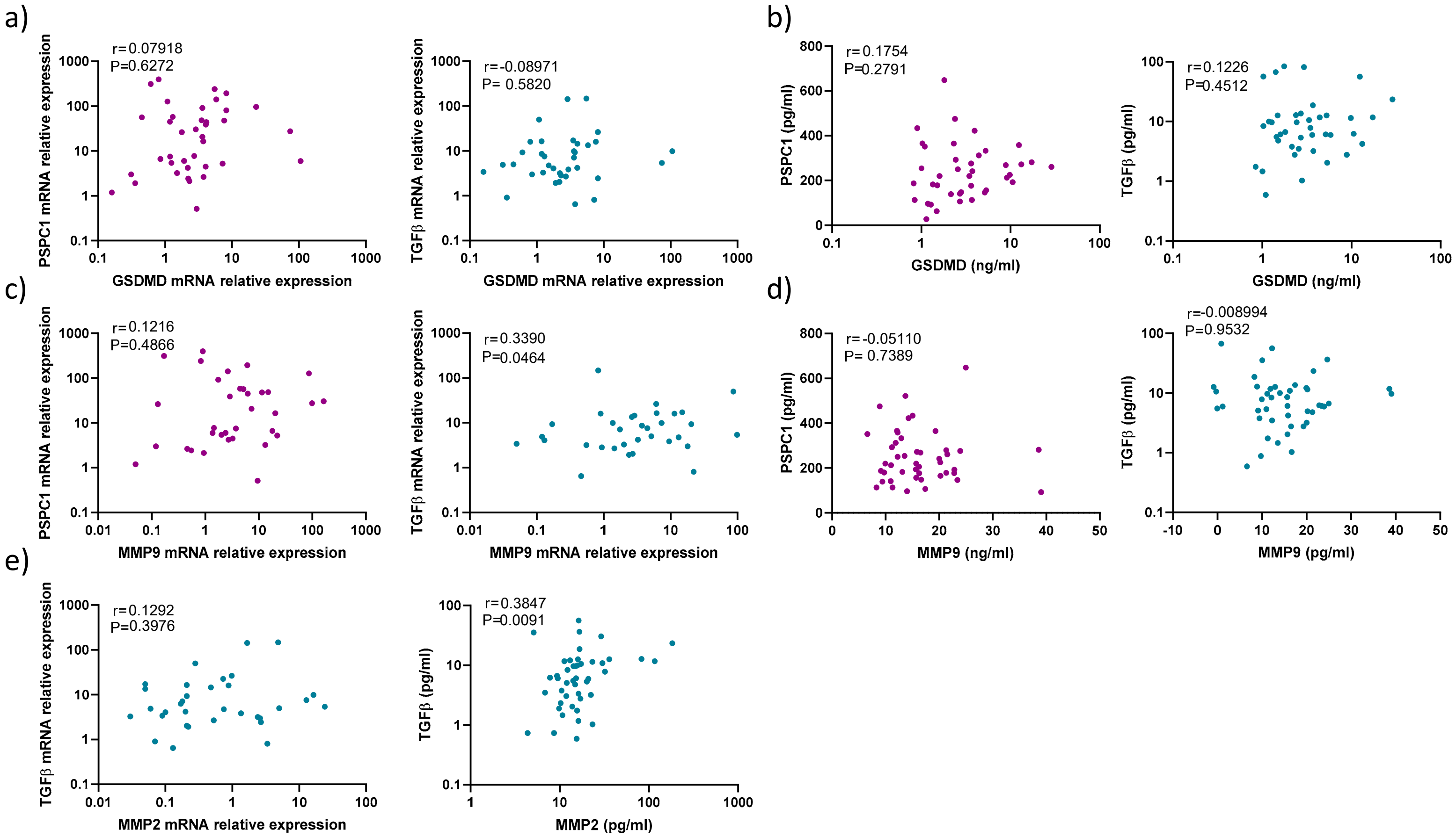

Figure S2. The PSPC1 and TGFβ expression correlations with GSDMD and MMP9

Correlation between GSDMD with PSPC1 and TGFβ mRNA expression in monocytes (a) and protein level from plasma (b) from severe OSA patients (n=40) that were randomly selected. Correlation between MMP9 with PSPC1 and TGFβ mRNA in monocytes (c, n=35) and protein level from plasma (d, n=45) from severe OSA patients that were randomly selected. Correlation between MMP2 with TGFβ mRNA in monocytes (c, n=45) and protein level from plasma (d, n=45)

from severe OSA patients that were randomly selected. Pearson correlation coefficients (R) and p-values are shown.

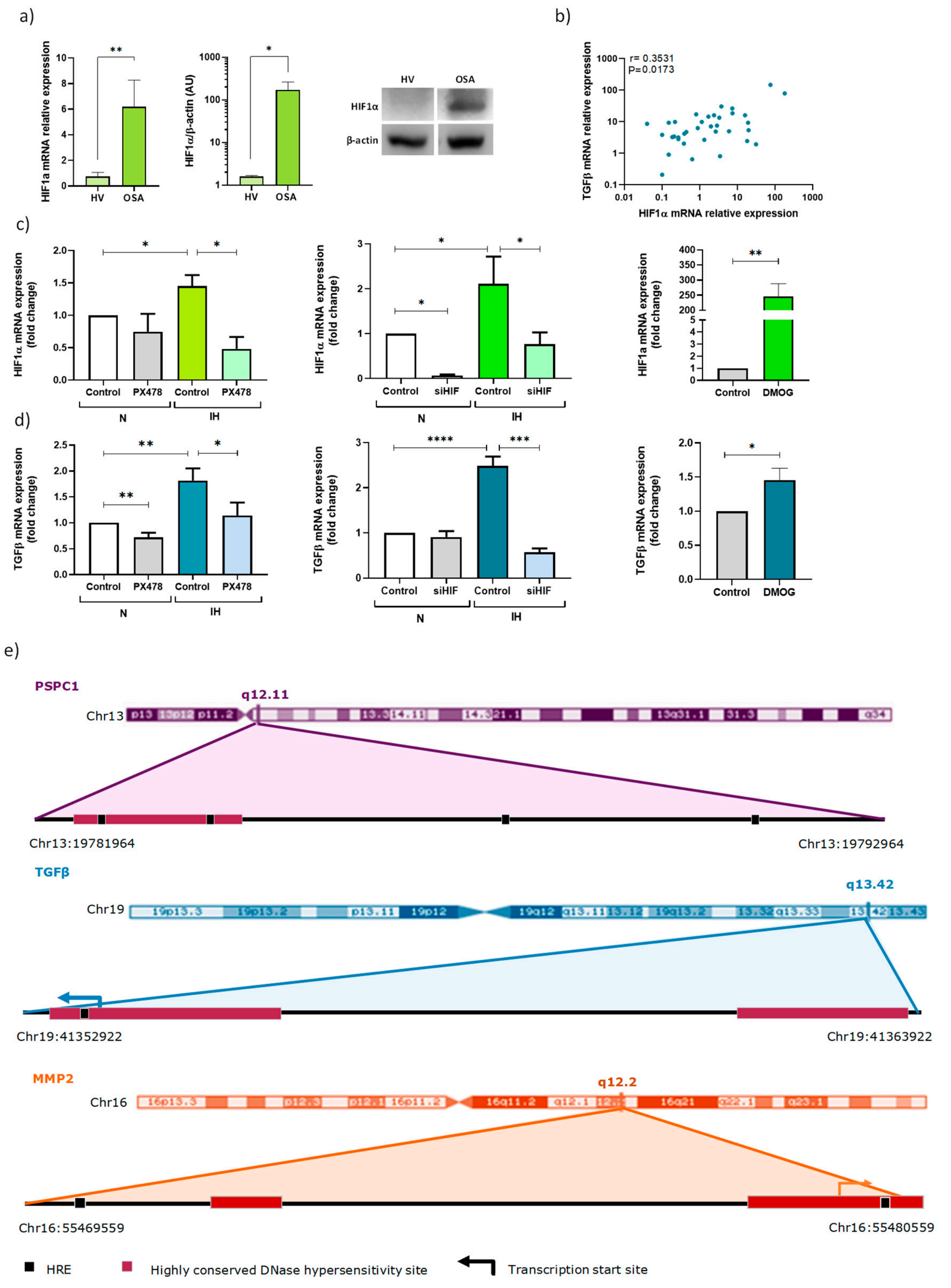

Figure S3. Hypoxemia is associated with TGFβ and MMP2 expression in OSA monocytes.

a) Comparison of HIF1α mRNA expression in monocytes from healthy volunteers (HV, n=20) and patients with severe OSA (n=41) (left panel). Representative western blot of monocytes from HV (n=3) and OSA (n=3) of HIF-1α and β-actin expression analysis. (right panel).

b) Correlation between HIF1α and TGFβ mRNA expression on monocytes from randomly selected OSA patients (n=40). Pearson correlation coefficients (r) and P-values are shown.

(c) HIF1α and (d) TGFβ mRNA expression estimation by qPCR in monocytes from HV subjects (n=7) treated with a specific inhibitor of HIF1α PX478 30μM or not and exposed to IH or normoxia conditions for 16 hours. (left panel), monocytes from HV subjects (n=3) treated with or not siHIF1α and exposed to IH or normoxia conditions for 16 hours (middle panel), monocytes from HV subjects (n=3) treated or not with DMOG for 2 hours (right panel). Comparisons between groups were performed by Two-way-ANOVA. Error bars: SEM.*:p<0.05, and **:p<0.01 versus non treated cells are shown.

e) Schematic representation of the in-silico analysis of the potential HRE binding sites, in the PSPC1, TGFβ and MMP2 promoter sequence (Ensemble reference sequence ENS), based on the consensus sequence. Location of these and HREs are shown. Data obtained from TRANSFAC® data base.

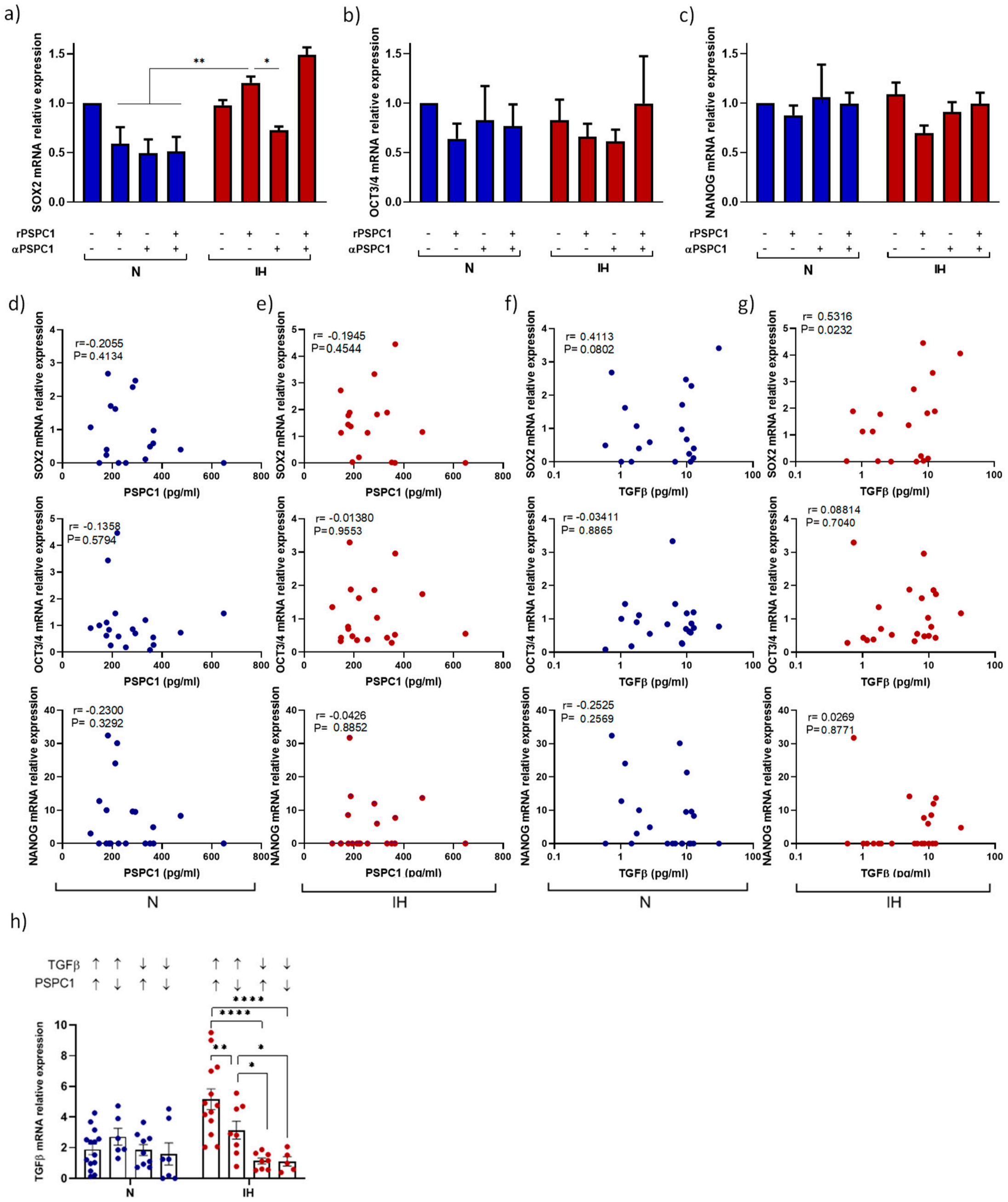

Figure S4. PSPC1 expression in OSA patients increase the effect on CSC.

The C-8161 melanoma cell line was cultured under normoxia and intermittent hypoxia (IH) conditions with rPSPC1 protein (2,5ng/ml) with or without α-PSPC1 (1ug/ml) during 16h. Then, melanoma cells were harvested to analyze the SOX2 (a), OCT3/4 (b) and NANOG (c) mRNA expression by qPCR. Comparisons between groups were performed by Two-way-ANOVA. Error bars: SEM.*:$p<0.05$ **:$p<0.01$ versus treated group respectively shown.

The melanoma cell line was cultured under IH and normoxia conditions with plasma from randomly selected severe OSA patients (10% concentration) during 16h. Then, melanoma cells were harvested to analyze the SOX2, OCT3/4 and NANOG mRNA expression by qPCR analysis. The correlation between PSPC1 protein concentrations from OSA plasma added to cell culture with SOX2, OCT3/4 and NANOG mRNA expression from melanoma cells cultured in normoxia (d) and IH (e) conditions are shown. The correlation between TGFβ protein concentrations from OSA plasma added to cell culture with SOX2, OCT3/4 and NANOG mRNA expression from melanoma cells cultured in normoxia (f) and IH (g) conditions is shown. Pearson correlation coefficients (r) and

P-values are shown.

h) The melanoma cell line was cultured under normoxia and IH conditions with plasma from OSA patients (10% concentration) during 16h. OSA plasma was selected according to the PSPC1 and TGFβ protein concentration; we performed four groups, PSPC1-high (200-2000pg/ml) with TGFβ-high (5-100pg/ml) as group 1, PSPC1-low (30-200pg/ml) with TGFβ-high as group 2, PSPC1-high (200-2000pg/ml) with TGFβ-low (0-5pg/ml) as group 3, PSPC1-low (30-200pg/ml) with TGFβ-low(0-5pg/ml) as group 4. Then, melanoma cells were harvested to analyze the mRNA expression by qPCR analysis. The TGFβ mRNA expression was analyzed by qPCR. The comparison between groups was performed with two-way ANOVA. Error bars: SEM. *:p<0.05, **:p<0.01, ****:p<0.0001 versus treated group respectively shown.

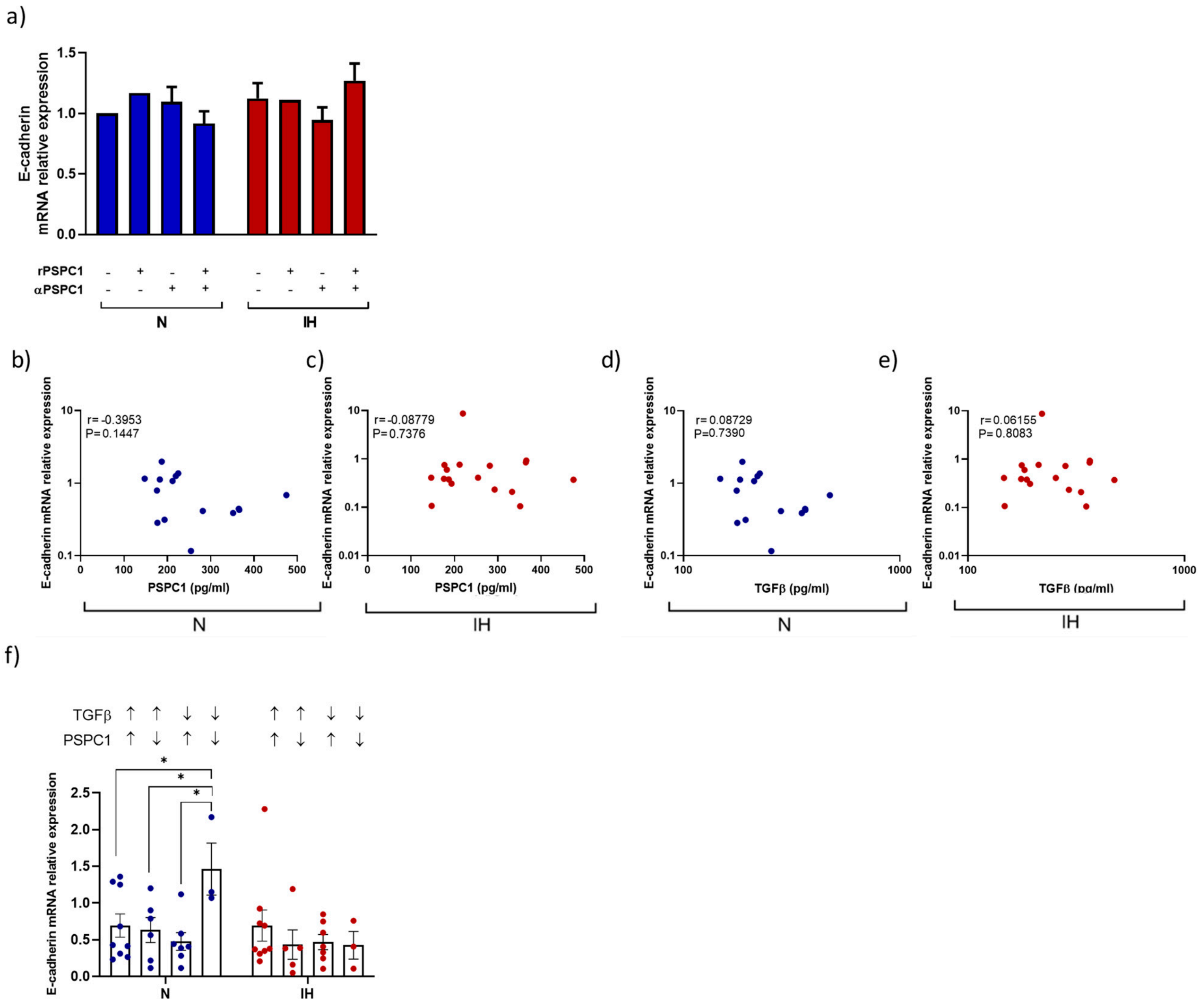

Figure S5. PSPC1 and TGFβ expression in OSA patients effect on E-cadherin expression.

a) The C-8161 melanoma cell line was cultured under normoxia and intermittent hypoxia (IH) conditions with rPSPC1 protein (2,5ng/ml) with or without α-PSPC1 (1ug/ml) during 16h. Then, melanoma cells were harvested to analyze the E-cadherin Comparisons between groups were performed by Two-way-ANOVA. Error bars: SEM.*:p<0.05 **:p<0.01 versus treated group respectively shown.

The melanoma cell line was cultured under IH and normoxia conditions with plasma from randomly selected severe OSA patients (10% concentration) during 16h. Then, melanoma cells were harvested to analyze the E-cadherin mRNA expression by qPCR analysis. The correlation between PSPC1 protein concentrations from OSA plasma added to cell culture with-cadherin mRNA expression from melanoma cells cultured in normoxia (b) and IH (c) conditions are shown. The correlation between TGFβ protein concentrations from OSA plasma added to cell culture with E-cadherin mRNA expression from melanoma cells cultured in normoxia (d) and IH (e) conditions is shown. Pearson correlation coefficients (r) and P-values are shown.

f) The melanoma cell line was cultured under normoxia and IH conditions with plasma from OSA patients (10% concentration) during 16h. OSA plasma was selected according to the PSPC1 and TGFβ protein concentration; we performed four groups, PSPC1-high (200-2000pg/ml) with TGFβ-high (5-100pg/ml) as group 1, PSPC1-low (30-200pg/ml) with TGFβ-high as group 2, PSPC1-high (200-2000pg/ml) with TGFβ-low (0-5pg/ml) as group 3, PSPC1-low (30-200pg/ml) with TGFβ-low(0-5pg/ml) as group 4. Then, melanoma cells were harvested to analyze the E-cadherin mRNA expression by qPCR analysis. The comparison between groups was performed with two-way ANOVA. Error bars: SEM. *:p<0.05

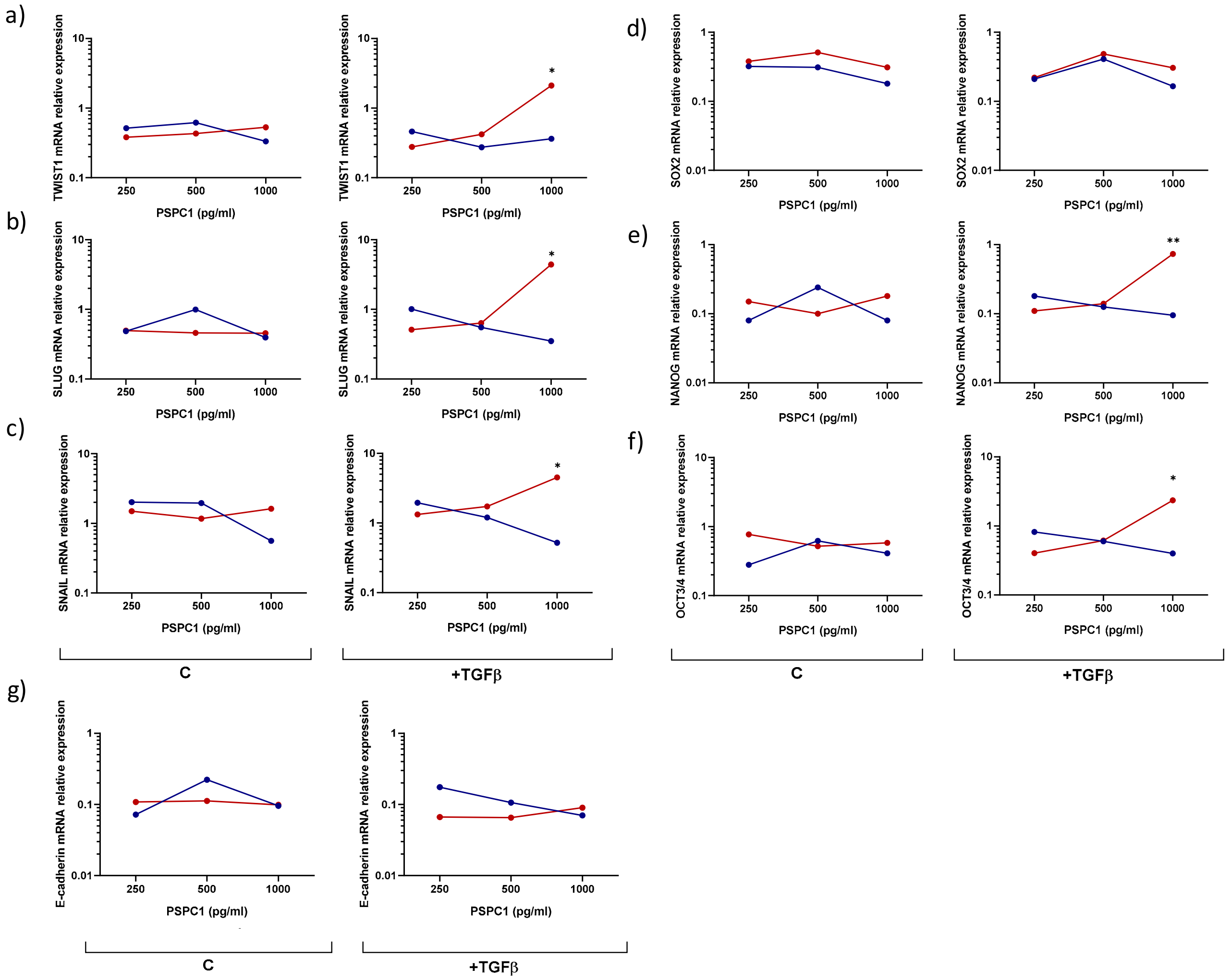

Figure S6. rPSPC1 effect on EMT.

The melanoma cell line (C-8161) was cultured under normoxia or IH conditions with different PSPC1 concentrations and with or without TGFβ during 16h. Then, melanoma cells were harvested to analyze the mRNA expression by qPCR analysis. a) TWIST b) SLUG c) SNAIL d) SOX2 e) NANOG, f) OCT3/4 g) E-cadherin mRNA expressions are shown. Mean values for two different experiments are shown. The comparison between groups was performed with two-way ANOVA. *:$p<0.05$ **:$p<0.01$, Mean values for two different experiments are shown.
